# AncestryGrapher toolkit: Python command-line pipelines to visualize global- and local- ancestry inferences from the RFMIX version 2 software

**DOI:** 10.1093/bioinformatics/btae616

**Published:** 2024-10-16

**Authors:** Alessandro Lisi, Michael C Campbell

**Affiliations:** Department of Biological Sciences (Human and Evolutionary Biology Section), University of Southern California, Los Angeles, CA 90089, United States; Department of Biological Sciences (Human and Evolutionary Biology Section), University of Southern California, Los Angeles, CA 90089, United States

## Abstract

**Summary:**

Admixture is a fundamental process that has shaped levels and patterns of genetic variation in human populations. RFMIX version 2 (RFMIX2) utilizes a robust modeling approach to identify the genetic ancestries in admixed populations. However, this software does not have a built-in method to visually summarize the results of analyses. Here, we introduce the AncestryGrapher toolkit, which converts the numerical output of RFMIX2 into graphical representations of global and local ancestry (i.e. the per-individual ancestry components and the genetic ancestry along chromosomes, respectively).

**Results:**

To demonstrate the utility of our methods, we applied the AncestryGrapher toolkit to visualize the global and local ancestry of individuals in the North African Mozabite Berber population from the Human Genome Diversity Panel. Our results showed that the Mozabite Berbers derived their ancestry from the Middle East, Europe, and sub-Saharan Africa (global ancestry). We also found that the population origin of ancestry varied considerably along chromosomes (local ancestry). For example, we observed variance in local ancestry in the genomic region on Chromosome 2 containing the regulatory sequence in the *MCM6* gene associated with lactase persistence, a human trait tied to the cultural development of adult milk consumption. Overall, the AncestryGrapher toolkit facilitates the exploration, interpretation, and reporting of ancestry patterns in human populations.

**Availability and implementation:**

The AncestryGrapher toolkit is free and open source on https://github.com/alisi1989/RFmix2-Pipeline-to-plot.

## 1 Introduction

Admixture, defined as gene flow between previously diverged source populations leading to new populations with ancestry from multiple sources, has been an important contributor to levels and patterns of genetic variation in humans (Rudan 2006, 1000 Genomes Project Consortium 2012, Hellenthal *et al.* 2014, Gurdasani *et al.* 2015, Uren *et al.* 2016, Korunes and Goldberg 2021). For example, a number of studies have shown that regions of the modern human genome have originated from archaic hominins, such as Neanderthals and Denisovans (Qin and Stoneking 2015, Sánchez-Quinto and Lalueza-Fox 2015, Sankararaman *et al.* 2016, Wall and Yoshihara Caldeira Brandt 2016, Browning *et al.* 2018, Vyas and Mulligan 2019, Koller *et al.* 2022, Vespasiani *et al.* 2022, Witt *et al.* 2023). Other analyses have also revealed that admixture among modern human populations has occurred over millennia, leading to complex patterns of genomic diversity (Campbell and Tishkoff 2008, Campbell *et al.* 2014, Bekada *et al.* 2015, Arauna *et al.* 2017, Korunes and Goldberg 2021, Gopalan *et al.* 2022).

Admixture in populations can be inferred by examining genetic ancestry at two levels: global and local (Tan and Atkinson 2023). Global ancestry refers to the proportion of an individual’s genome that originated from distinct source populations, while local ancestry refers to an individual’s genetic ancestry from source populations at particular chromosomal locations (Liu *et al.* 2013, Goli *et al.* 2024). To characterize patterns of admixture, several computational methods have been developed to infer global ancestry (Pritchard *et al.* 2000, Hoggart *et al.* 2004, Patterson *et al.* 2004, Alexander *et al.* 2009, Lawson *et al.* 2012, Liu *et al.* 2013, Hellenthal *et al.* 2014, Raj *et al.* 2014, Wangkumhang *et al.* 2022) and local ancestry along chromosomes in individuals (Falush *et al.* 2003, Tang *et al.* 2005, 2006, Sankararaman *et al.* 2008a, 2008b, Sundquist *et al.* 2008a, 2008b; Pasaniuc *et al.* 2009, Price *et al.* 2009, Bryc *et al.* 2010, Baran *et al.* 2012, Brisbin *et al.* 2012, Churchhouse and Marchini 2013, Maples *et al.* 2013, Durand *et al.* 2014, Guan 2014, Dias-Alves *et al.* 2018, Salter-Townshend and Myers 2019, Montserrat *et al.* 2020, Molinaro *et al.* 2021, Browning *et al.* 2023). Of these methods, RFMix v.1.5.4 is considered to be state-of-the-art in estimating ancestry in complex admixture scenarios (Daya *et al.* 2014, Uren *et al.* 2020, Hilmarsson *et al.* 2021). Furthermore, an updated version of this software—called RFMIX version 2 (RFMIX2) (https://github.com/slowkoni/rfmix)—was recently developed and is argued to be superior to the previous version (Carrot-Zhang *et al.* 2021). However, there is currently no straightforward way to visualize the global and local ancestry results from RFMIX2, which is important for the exploration, interpretation, and reporting of ancestry patterns in human populations. To address this problem, we developed computational tools to depict both global and local ancestry, as inferred by RFMIX2, in individuals from admixed groups.

## 2 Implementation

Inferences of genetic ancestry are informative for determining the population origins of alleles associated with traits, including disease susceptibility (Freedman *et al.* 2006, Cheng *et al.* 2009, Daya *et al.* 2014) and for understanding the genetic history of admixed populations (most notably, past migration events) (Daya *et al.* 2014, Uren *et al.* 2020; Gopalan *et al.* 2022; Browning *et al.* 2023).

**Figure 1.**
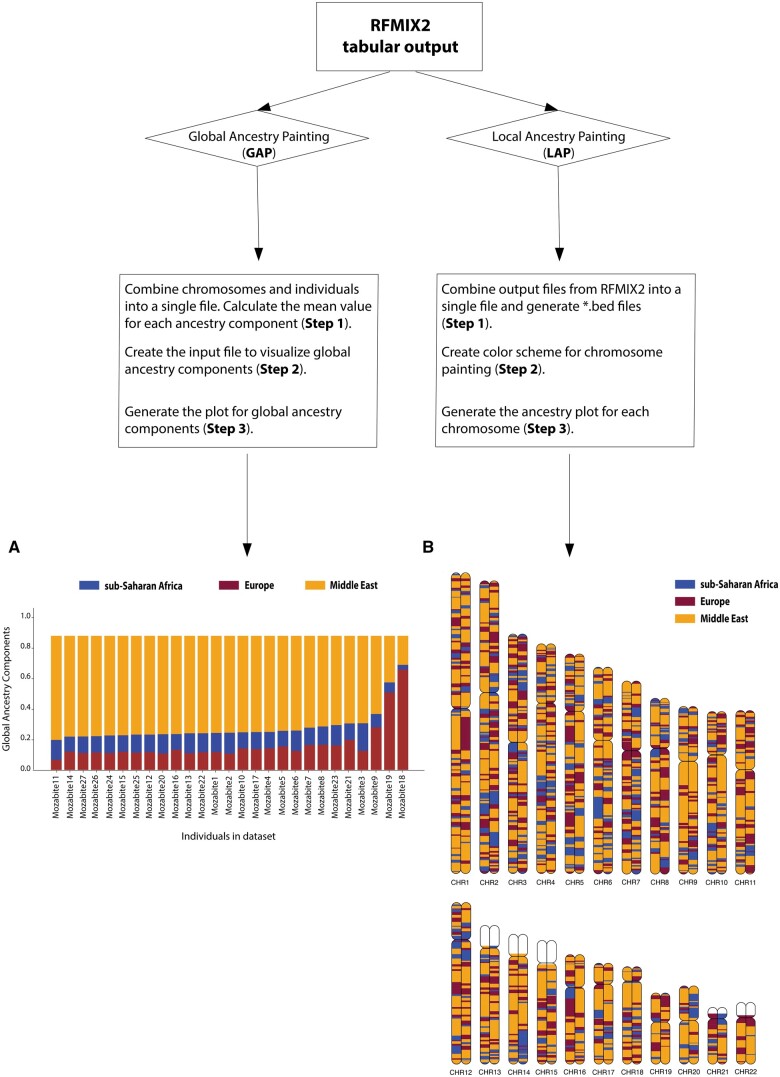
Workflow of the AncestryGrapher toolkit. AncestryGrapher consists of two pipelines: (i) GAP and (ii) LAP that run in a command-line Terminal. GAP combines individuals and chromosomes into a single file, calculates mean ancestry components across chromosomes per individual, and visualizes their ancestry in a bar plot (i.e., global ancestry). Panel A shows the plotted mean ancestry components per individual in the Mozabite population from the Human Genome Diversity Panel (Rosenberg *et al.* 2002; Li *et al.* 2008; Bergström *et al.* 2020). Individuals have been arranged by the Middle Eastern ancestry component from largest to smallest. LAP combines the output from RFMIX2 into a single file, creates a color scheme for ancestry painting, and plots the ancestry along chromosomes (i.e., local ancestry). Panel B shows the local ancestry components along diploid Chromosomes 1 through 22 for a Mozabite individual. In Panels A and B, the orange color indicates Middle Eastern ancestry; purple represents European ancestry; and blue signifies sub-Saharan African ancestry. The white color in Panel B represents regions of the genome with unknown ancestry (i.e., the reference ancestry is not present) and/or these regions contain missing SNP data.

The AncestryGrapher toolkit is a very powerful package for displaying the broad ancestry proportions originating from source populations that individuals possess (i.e. global ancestry), which is helpful when selecting cohorts of individuals with either a high or low level of a particular ancestry for admixture mapping analyses (Caliebe *et al.* 2022; Shriner 2023). Furthermore, the AncestryGrapher toolkit can visualize the genetic ancestry within genomic coordinates along chromosomes. allowing for the efficient identification of the population origins of alleles (i.e. local ancestry) in anthropological studies (Gravel 2012, Secolin *et al.* 2019). The AncestryGrapher toolkit enables users to visualize these levels of ancestry with two distinct pipelines, Global Ancestry Painting (GAP) and Local Ancestry Painting (LAP; Fig. 1).

### 2.1 Global Ancestry Painting

The Global Ancestry Painting (GAP) pipeline consists of three separate Python scripts: (i) RFMIX2ToBed4GAP.py; (ii) BedToGAP.py; and (iii) GAP.py.

#### 2.1.1 Step 1: Combine the RFMIX2 output files for all chromosomes and individuals into a single file

The RFMIX2 software generates a *.rfmix.Q (global ancestry information) output file for each chromosome per individual in a given dataset. The RFMIX2ToBed4GAP.py script will combine these separate files into a single file and then calculate a mean value for each inferred ancestry component across chromosomes per individual. If users wish to sort individuals by a particular mean ancestry component (from largest to smallest), they can accomplish this task by implementing the “--sort-ancestry” flag.

#### 2.1.2 Step 2: Create the input file for GAP to visualize the global ancestry components

The output file generated in Step 1 will serve as the input file for BedToGAP.py. Here, users can select up to 10 distinct colors to represent ancestry (one color for each mean ancestry component).

#### 2.1.3 Step 3: Generate the plot for global ancestry components

In this step, users will run the GAP.py script to generate an output file with mean ancestry components in a bar plot for all individuals, together with a legend of ancestry origin and the name of each individual in the dataset, in either “pdf” or “svg” format (Fig. 1A).

### 2.2 Local Ancestry Painting

The Local Ancestry Painting (LAP) pipeline consists of three separate Python scripts: (i) RFMIX2ToBed.py; (ii) BedToLAP.py; and (iii) LAP.py.

#### 2.2.1 Step 1: Combine the output files from RFMIX2 into a single file and generate *.bed files

RFMix2 generates a *.msp.tsv (local ancestry information) output file for each chromosome (e.g. 1–22) for a given individual. RFMIX2ToBed.py will combine these chromosomes into a single file that contains header and ancestry information for all chromosomes for each individual in the dataset. RFMIX2ToBed.py will also generate two *.bed files (*_hap1.bed and *_hap2.bed) per individual, corresponding to maternal and paternal chromosomes in diploid organisms (with one set of chromosomes in each *.bed file).

#### 2.2.2 Step 2: Create the color scheme for ancestry painting along chromosomes

Here, the output files from Step 1 will serve as the input files in this step. BedToLAP.py will assign a default color to each ancestry component inferred by RFMIX2 (a maximum of 10 colors will be generated, one for each ancestry component). Alternatively, users can select up to 10 distinct colors with the “--ancestry” flag. BedToLAP.py can also highlight a specific gene or genomic region of interest on a chromosome with additional parameters.

#### 2.2.3 Step 3: Generate the ancestry plot for each chromosome for a given individual

The resulting output files, generated using the LAP.py script, will contain ancestry-informative karyograms along with a legend of ancestry origin and other identifiers (Fig. 1B). The images within the output files will have 4k resolution (4210 × 1663) and can be edited in Adobe Illustrator or Inkscape.

## 3 Results and discussion

We have developed the AncestryGrapher toolkit to visually summarize the output of the RFMIX2 software. To demonstrate the utility of our computational pipelines, we applied the GAP and LAP algorithms to plot the inferred ancestry of individuals in the North African Mozabite Berber population from the Human Genome Diversity Panel (Rosenberg *et al.* 2002, Li *et al.* 2008, Bergström *et al.* 2020). Our results showed that individuals in this population have distinct levels of Middle Eastern, European, and sub-Saharan African ancestry (Fig. 1), consistent with prior studies (Henn *et al.* 2012, Bekada *et al.* 2015, Arauna *et al.* 2017, Silva *et al.* 2021).

In addition, we detected striking patterns of local ancestry along individual chromosomes. For this analysis, we focused our attention on the well-studied regulatory region in intron 13 of the *MCM6* gene on Chromosome 2, which contains single nucleotide polymorphisms associated with lactase persistence—namely, C/T_−13910_ (rs4988235), C/G_−13907_ (rs41525747), T/G_−13915_ (rs41380347), T/G_−14009_ (rs869051967), and G/C_−14010_ (rs145946881) (Enattah *et al.* 2002, Ingram *et al.* 2007, 2009, Tishkoff *et al.* 2007, Hassan *et al.* 2016, Anguita-Ruiz *et al.* 2020, Campbell and Ranciaro 2021). Prior studies also have shown that these functional sites have been the targets of Darwinian selection in Middle Eastern, European, and sub-Saharan African populations with a history of dairying (Bersaglieri *et al.* 2004, Tishkoff *et al.* 2007, Enattah *et al.* 2008, Gerbault *et al.* 2009, Itan *et al.* 2009, Jones *et al.* 2013, Macholdt *et al.* 2014, Ranciaro *et al.* 2014, Scheinfeldt *et al.* 2019, Vicente *et al.* 2019, Hollfelder *et al.* 2020). Based on our LAP plots, we found that alleles in the *MCM6* regulatory sequence in the Mozabite Berber population sit on a chromosomal segment on Chromosome 2 originating from the Middle East, Europe, and/or sub-Saharan Africa. More specifically, alleles in the *MCM6* regulatory region were found to sit in a chromosomal segment originating from the Middle East on 53.7% of chromosomes, while alleles in the same region had ancestry backgrounds originating from Europe and sub-Saharan Africa on 31.5% and 14.8% of chromosomes, respectively, in the Mozabite Berbers (Supplementary Fig. S1). Thus, genetic variation in the *MCM6* regulatory region was likely introduced into this population through admixture, which we visually captured using the LAP pipeline. For comparison, we also show GAP and LAP results for different populations with a history of geographic and cultural isolation, namely the Finnish (Europe) and the Bedouin (Middle East) in Supplementary Figs S2 and S3, respectively.

In summary, the AncestryGrapher toolkit has the capability to calculate the mean ancestry components based on RFMIX2 output files and provide a graphical display of this global ancestry for each individual in a given dataset. Furthermore, this package can convert the ancestry tracts from RFMIX2 into colors representing ancestry along chromosomes. While these strengths make AncestryGrapher a powerful tool for exploring, interpreting, and reporting results, it is also important to note the limitations of our approaches and the RFMIX2 software. Although there is no restriction on the number of target individuals that can be visualized with the AncestryGrapher toolkit, the GAP and LAP pipelines can at most assign colors to a maximum of 10 ancestry components (or source populations). Consequently, ancestry components above the 10th one will not be displayed. Regarding RFMIX2, this software is especially effective when users have included populations that have contributed genetic ancestry to the admixed population of interest. In addition, these RFMIX2 analyses are powered by well-characterized genetic maps for SNP loci along chromosomes. Thus, RFMIX2 should only be applied to datasets with valid reference populations based on *a priori* information and reliable genetic maps. Assuming RFMIX2 is used correctly, the AncestryGrapher toolkit is highly beneficial for graphically summarizing genetic patterns in complex populations supporting anthropological and biomedical research (for more detailed information about RFMIX2 and other ancestry methods, please see Supplementary Table S1).

## Supplementary Material

btae616_Supplementary_Data

## Data Availability

The data underlying this article are available in the Human Genome Diversity Panel at https://ngs.sanger.ac.uk//production/hgdp/hgdp_wgs.20190516/statphase/ and in the 1000 Genomes Consortium Project at https://www.internationalgenome.org/data-portal/population/FIN.
