# Supplementary material for: AncestryGrapher toolkit: Python command-line pipelines to visualize global- and local- ancestry inferences from the RFMIX version 2 software
Source: Bioinformatics. 2024 Oct 16;40(11):btae616. doi: 10.1093/bioinformatics/btae616 (PMC11534077; doi:10.1093/bioinformatics/btae616)
Supplement: btae616_Supplementary_Data [file btae616_supplementary_data.pdf]

Supplementary Table Legend

Table S1— Table summarizing key characteristics of different admixture software. Here, we summarize the differences and similarities of various approaches for estimating genetic ancestry, particularly their methods as described in the original publication, the data to which they may be applied, the format of input files, and the parameters that must be specified by users. Concerning the data, there are limits on the number and type of SNPs for some software. Specifically, STRUCTURE can accommodate ~10,000 genomic SNPs (small); and ANCESTRYMAP can accommodate 1,000-3,000 SNPs (small). For ADMIXMAP, a subset of SNPs (at least 1,000) should be ancestry informative markers (AIMS). LAMP-LD can accommodate ~50,000 SNPs. Medium to large refers to datasets containing >400,000 SNPs. In this Table, we also indicate the level(s) of ancestry that each software was mainly designed to estimate. Notably, RFMIX2 (shaded) can harness prior information to estimate global and local ancestry, such as haplotype phase and the use of genetic locations of SNPs (genetic maps), allowing for the increased accuracy of ancestry inference. The full terms for abbreviations, under the ancestry inference method header, are given as follows: MCMC (Markov Chain Monte Carlo), HMM (Hidden Markov Model), EM (Expectation-Maximization), ML (Maximum Likelihood), MHMM (Markov Hidden Markov Model), PCA (Principal Component Analysis), CEM (Classification Expectation- Maximization), and RF (Random Field). *While LAMP-LP uses a constant recombination map by default, the authors of this software indicated that LAMP-LP can scale the physical positions of SNPs with any recombination map of loci.

Supplementary Figure Legends

Figure S1: Local ancestry along Chromosome 2 in the North African Mozabite Berber population from the Human Genome Diversity Panel. These plots show the local ancestry along Chromosome 2 together with a legend of ancestry origin and the name given to each individual in the Mozabite population from the Human Genome Diversity Panel (Rosenberg et al. 2002; Li et al. 2008; Bergström et al. 2020). The orange color indicates Middle Eastern ancestry; blue represents European ancestry; and red signifies ancestry originating from sub-Saharan Africa. The dashed horizontal line indicates the chromosomal segment containing the regulatory sequence in intron 13 of the *MCM6* gene associated with lactase persistence.

Figure S2: Global and local ancestry for a subset of individuals from the Finnish population in the 1000 Genomes Project. **Panel A** shows the mean ancestry components with the corresponding legend at the top for a subset of individuals from the Finnish population in the 1000 Genomes Project (The 1000 Genomes Consortium, 2012). **Panel B** shows the local ancestry along Chromosome 2 for the same individuals (as shown in Panel A) together with the chromosome number and a legend of ancestry origin. We also highlighted the regulatory sequence in intron 13 of the *MCM6* gene on this chromosome with a dashed horizontal line. In addition, the white color in the local ancestry plots, if it appears, represents regions of the genome with unknown ancestry (*i.e.*, the reference ancestry was not present in the RFMIX2 analysis) and/or these regions contain missing SNP data.

Figure S3: Global and local ancestry for a subset of individuals from the Bedouin population in the Human Genome Diversity Panel. **Panel A** shows the mean ancestry components with the corresponding legend at the top for a subset of individuals from the Bedouin population in the Human Genome Diversity Panel (Rosenberg et al. 2002; Li et al. 2008; Bergström et al. 2020). **Panel B** shows the local ancestry along Chromosome 2 for the same individuals (as shown in Panel A) together with the chromosome number and a legend of ancestry origin. We also highlighted the regulatory sequence in intron 13 of the *MCM6* gene on this chromosome with a dashed horizontal line. In addition, the white color in the local ancestry plot, if it appears, represents regions of the genome with unknown ancestry (*i.e.*, the reference ancestry was not present in the RFMIX2 analysis) and/or these regions contain missing SNP data.

Table S1

Software	Global Ancestry	Local Ancestry	Ancestry Inference Method	Phased data required	Genetic map required	Size of dataset (number of SNPs)	Number of reference populations allowed	References
STRUCTURE	✓	✗	MCMC	✗	✗	small	≥ 2	(Pritchard, Stephens, and Donnelly 2000). Falush et al. (2003) extended STRUCTURE to estimate local ancestry (Falush, Stephens, and Pritchard 2003).
fastSTRUCTURE	✓	✗	Variational Bayesian framework	✗	✗	medium-large	≥ 2	(Raj, Stephens, and Pritchard 2014)
GLOBETROTTER	✓	✗	HMM	✓	✓	medium-large	≥ 2	(Hellenthal et al. 2014); uses output from ChromoPainter
fastGLOBETROTTER	✓	✗	HMM	✓	✓	medium-large	≥ 2	(Wangkumhang, Greenfield, and Hellenthal 2022); uses output from ChromoPainter
ANCESTRYMAP	✓	✓	HMM	✗	✓	small		(Patterson et al. 2004)
ADMIXMAP	✓	✓	MCMC	✗	✓	small	≥ 2	(Hoggart et al. 2004)
FRAPPE	✓	✗	EM-ML	✗	✗	medium-large	≥ 2	(Tang et al. 2005)
ADMIXTURE	✓	✗	Block relaxation-ML	✗	✗	medium-large	≥ 2	(Alexander, Novembre, and Lange 2009)
SABER	✗	✓	MHMM	✓	✗	medium-large	≥ 2	(Tang et al. 2006)
ChromoPainter	✗	✓	HMM	✓	✓	medium-large	≥ 2	(Lawson et al. 2012)
HAPAA	✗	✓	nested-HMM	✓	✓	medium-large	≥ 2	(Sundquist et al. 2008)
SWITCH	✗	✓	HMM-EM	✓	✗	medium-large	≥ 2	(Sankararaman, Kimmel, et al. 2008)
SWITCH-MHMM	✗	✓	MHMM-EM	✓	✗	medium-large	≥ 2	(Sankararaman, Kimmel, et al. 2008)
WINPOP	✗	✓	clustering-majority vote	✗	✗	medium-large	≥ 2	(Pasaniuc et al. 2009)
HAPMIX	✗	✓	nested-HMM	✓	✓	medium-large	2	(Price et al. 2009)
PCAdmix	✗	✓	HMM-PCA	✓	✗	medium-large	≥ 2	(Bryc et al. 2010)
MULTIMIX	✗	✓	MCMC, EM, or CEM	✗	✗	medium-large	≥ 2	(Churchhouse and Marchini 2013)
LAMP	✗	✓	clustering-majority vote	✗	✗	medium-large	≥ 2	(Sankararaman, Sridhar, et al. 2008)
LAMP-LD	✗	✓	HMM	✓	✗*	small	2,3, or 5	(Baran et al. 2012)
RFMix	✗	✓	HMM-RF	✓	✗	medium-large	≥ 2	(Maples et al. 2013)
RFMIX2	✓	✓	HMM-RF	✓	✓	medium-large	≥ 2	https://github.com/slowkoni/rfmix
ELAI	✗	✓	two-layer HMM	✗	✗	medium-large	≥ 2	(Guan 2014)
Loter	✗	✓	HMM	✓	✗	medium-large	≥ 2	(Thomas Dias-Alves, Julien Mairal, Michael G B Blum 2018)
FLARE	✗	✓	HMM	✓	✓	medium-large	≥ 2	(Browning, Waples, and Browning 2023)
MOSAIC	✗	✓	nested-HMM-EM	✓	✓	medium-large	≥ 2	(Salter-Townshend and Myers 2019)

References Cited

1. Alexander, David H., John Novembre, and Kenneth Lange. 2009. "Fast Model-Based Estimation of Ancestry in Unrelated Individuals." *Genome Research* 19 (9): 1655–64.
2. Baran, Yael, Bogdan Pasaniuc, Sriram Sankararaman, Dara G. Torgerson, Christopher Gignoux, Celeste Eng, William Rodriguez-Cintron, et al. 2012. "Fast and Accurate Inference of Local Ancestry in Latino Populations." *Bioinformatics (Oxford, England)* 28 (10): 1359–67.
3. Browning, Sharon R., Ryan K. Waples, and Brian L. Browning. 2023. "Fast, Accurate Local Ancestry Inference with FLARE." *The American Journal of Human Genetics* 110 (2): 326–35.
4. Bryc, Katarzyna, Adam Auton, Matthew R. Nelson, Jorge R. Oksenberg, Stephen L. Hauser, Scott Williams, Alain Froment, et al. 2010. "Genome-Wide Patterns of Population Structure and Admixture in West Africans and African Americans." *Proceedings of the National Academy of Sciences of the United States of America* 107 (2): 786–91.
5. Churchhouse, Claire, and Jonathan Marchini. 2013. "Multiway Admixture Deconvolution Using Phased or Unphased Ancestral Panels." *Genetic Epidemiology* 37 (1): 1–12.
6. Falush, Daniel, Matthew Stephens, and Jonathan K. Pritchard. 2003. "Inference of Population Structure Using Multilocus Genotype Data: Linked Loci and Correlated Allele Frequencies." *Genetics* 164 (4): 1567–87.
7. Guan, Yongtao. 2014. "Detecting Structure of Haplotypes and Local Ancestry." *Genetics* 196 (3): 625–42.
8. Hellenthal, G., G. B. J. Busby, G. Band, J. F. Wilson, C. Capelli, D. Falush, and S. Myers. 2014. "A Genetic Atlas of Human Admixture History." *Science (New York, N.Y.)* 343 (6172): 747–51.
9. Hoggart, C. J., M. D. Shriver, R. A. Kittles, D. G. Clayton, and P. M. McKeigue. 2004. "Design and Analysis of Admixture Mapping Studies." *The American Journal of Human Genetics* 74 (5): 965–78.
10. Lawson, Daniel John, Garrett Hellenthal, Simon Myers, and Daniel Falush. 2012. "Inference of Population Structure Using Dense Haplotype Data." *PLoS Genetics* 8 (1): e1002453.
11. Maples, Brian K., Simon Gravel, Eimear E. Kenny, and Carlos D. Bustamante. 2013. "RFMix: A Discriminative Modeling Approach for Rapid and Robust Local-Ancestry Inference." *The American Journal of Human Genetics* 93 (2): 278–88.
12. Pasaniuc, Bogdan, Sriram Sankararaman, Gad Kimmel, and Eran Halperin. 2009. "Inference of Locus-Specific Ancestry in Closely Related Populations." *Bioinformatics (Oxford, England)* 25 (12): i213-21.
13. Patterson, Nick, Neil Hattangadi, Barton Lane, Kirk E. Lohmueller, David A. Hafler, Jorge R. Oksenberg, Stephen L. Hauser, et al. 2004. "Methods for High-Density Admixture Mapping of Disease Genes." *The American Journal of Human Genetics* 74 (5): 979–1000.
14. Price, Alkes L., Arti Tandon, Nick Patterson, Kathleen C. Barnes, Nicholas Rafaels, Ingo Ruczinski, Terri H. Beaty, Rasika Mathias, David Reich, and Simon Myers. 2009. "Sensitive Detection of Chromosomal Segments of Distinct Ancestry in Admixed Populations." *PLoS Genetics* 5 (6): e1000519.
15. Pritchard, J. K., M. Stephens, and P. Donnelly. 2000. "Inference of Population Structure Using Multilocus Genotype Data." *Genetics* 155 (2): 945–59.
16. Raj, Anil, Matthew Stephens, and Jonathan K. Pritchard. 2014. "FastSTRUCTURE: Variational Inference of Population Structure in Large SNP Data Sets." *Genetics* 197 (2): 573–89.
17. Salter-Townshend, Michael, and Simon Myers. 2019. "Fine-Scale Inference of Ancestry Segments without Prior Knowledge of Admixing Groups." *Genetics* 212 (3): 869–89.
18. Sankararaman, Sriram, Gad Kimmel, Eran Halperin, and Michael I. Jordan. 2008. "On the Inference of Ancestries in Admixed Populations." *Genome Research* 18 (4): 668–75.
19. Sankararaman, Sriram, Srinath Sridhar, Gad Kimmel, and Eran Halperin. 2008. "Estimating Local Ancestry in Admixed Populations." *The American Journal of Human Genetics* 82 (2): 290–303.
20. Sundquist, Andreas, Eugene Fratkin, Chuong B. Do, and Serafim Batzoglou. 2008. "Effect of Genetic Divergence in Identifying Ancestral Origin Using HAPAA." *Genome Research* 18 (4): 676–82.
21. Tang, Hua, Marc Coram, Pei Wang, Xiaofeng Zhu, and Neil Risch. 2006. "Reconstructing Genetic Ancestry Blocks in Admixed Individuals." *The American Journal of Human Genetics* 79 (1): 1–12.
22. Tang, Hua, Jie Peng, Pei Wang, and Neil J. Risch. 2005. "Estimation of Individual Admixture: Analytical and Study Design Considerations." *Genetic Epidemiology* 28 (4): 289–301.

23. The 1000 Genomes Project Consortium. An integrated map of genetic variation from 1,092 human genomes. Nature 2012;491:56–65.
24. Thomas Dias-Alves, Julien Mairal, Michael G B Blum. 2018. "Loter: A Software Package to Infer Local Ancestry for a Wide Range of Species." *Molecular Biology and Evolution* 35 (9): 2318–26.
25. Wangkumhang, Pongsakorn, Matthew Greenfield, and Garrett Hellenthal. 2022. "An Efficient Method to Identify, Date, and Describe Admixture Events Using Haplotype Information." *Genome Research* 32 (8): 1553–64.

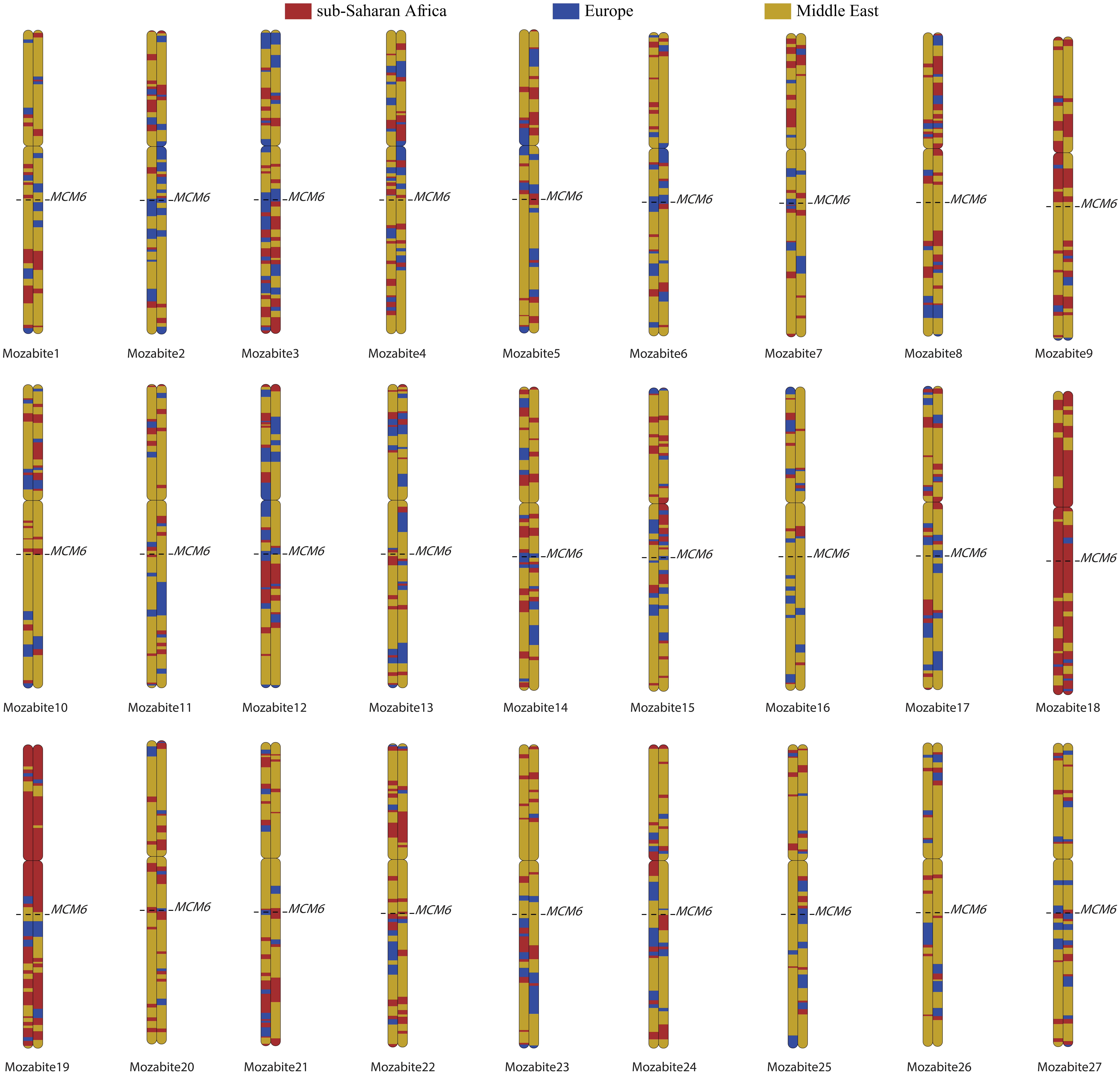
sub-Saharan Africa
Europe
Middle East
MCM6
Mozabite1
Mozabite2
Mozabite3
Mozabite4
Mozabite5
Mozabite6
Mozabite7
Mozabite8
Mozabite9
Mozabite10
Mozabite11
Mozabite12
Mozabite13
Mozabite14
Mozabite15
Mozabite16
Mozabite17
Mozabite18
Mozabite19
Mozabite20
Mozabite21
Mozabite22
Mozabite23
Mozabite24
Mozabite25
Mozabite26
Mozabite27

Figure S1

A.

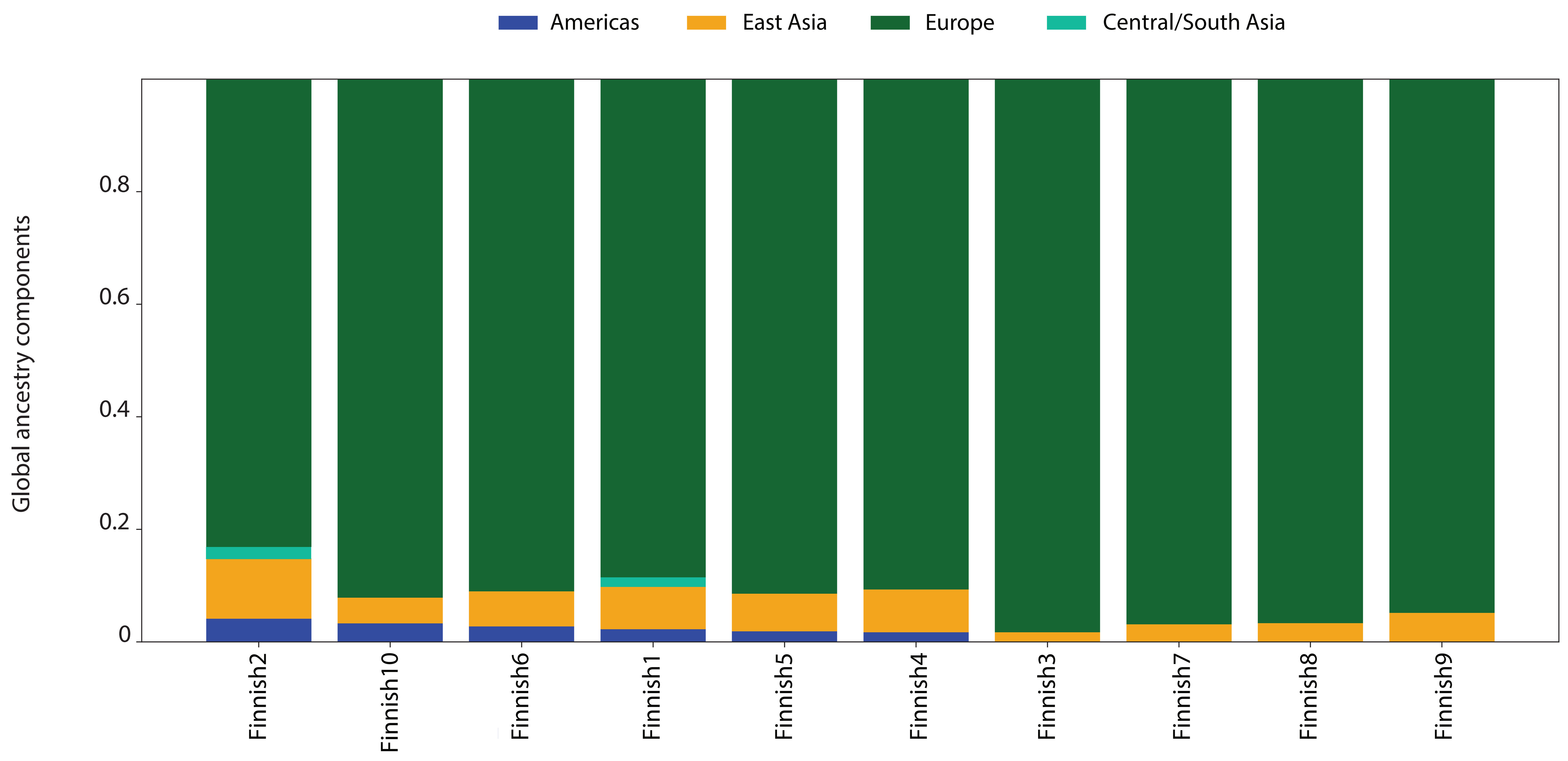
Americas
East Asia
Europe
Central/South Asia
Global ancestry components
0.8
0.6
0.4
0.2
0
Finnish2
Finnish10
Finnish6
Finnish1
Finnish5
Finnish4
Finnish3
Finnish7
Finnish8
Finnish9

B.

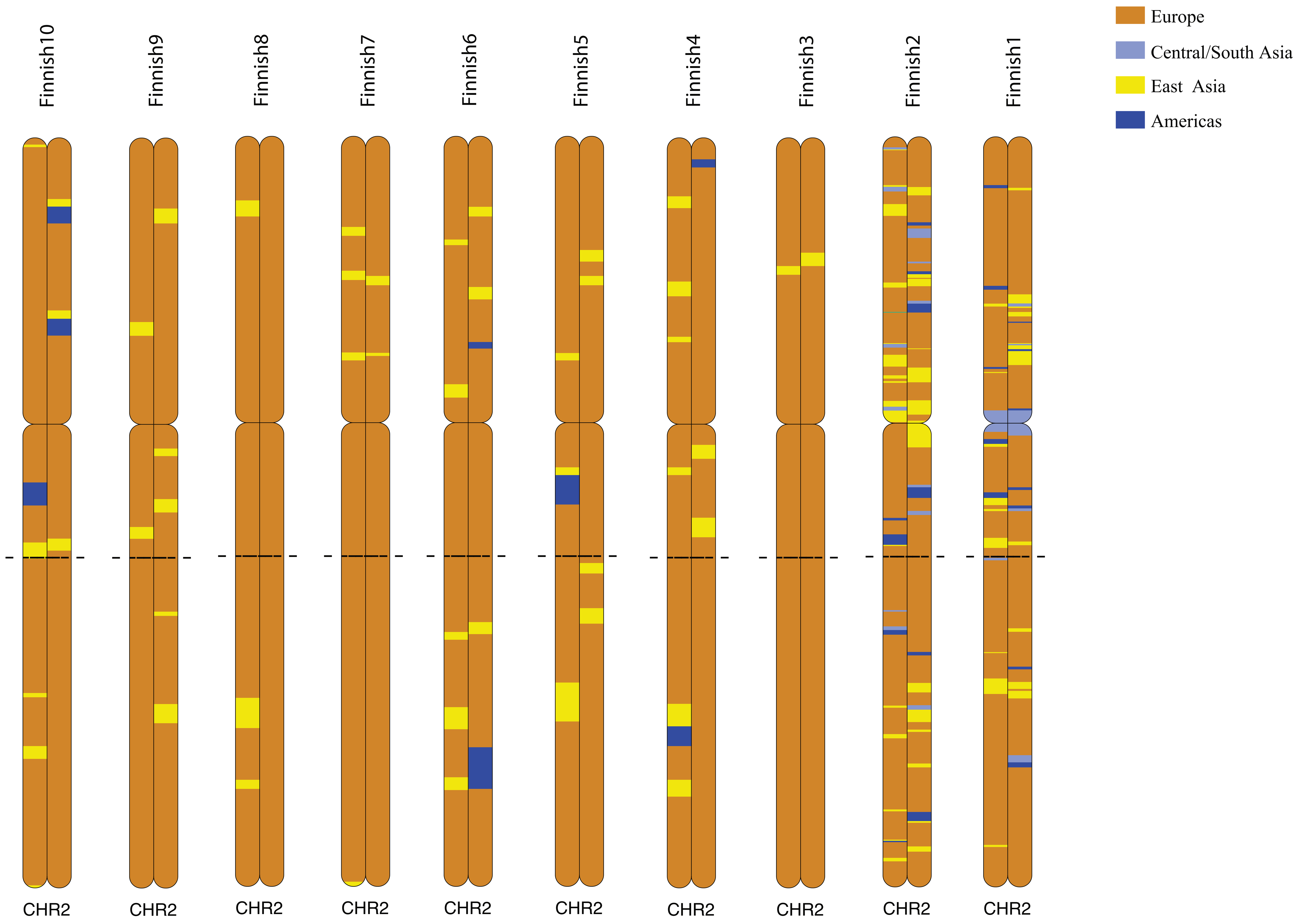
Finnish10
Finnish9
Finnish8
Finnish7
Finnish6
Finnish5
Finnish4
Finnish3
Finnish2
Finnish1
Europe
Central/South Asia
East Asia
Americas
CHR2

Figure S2

A.

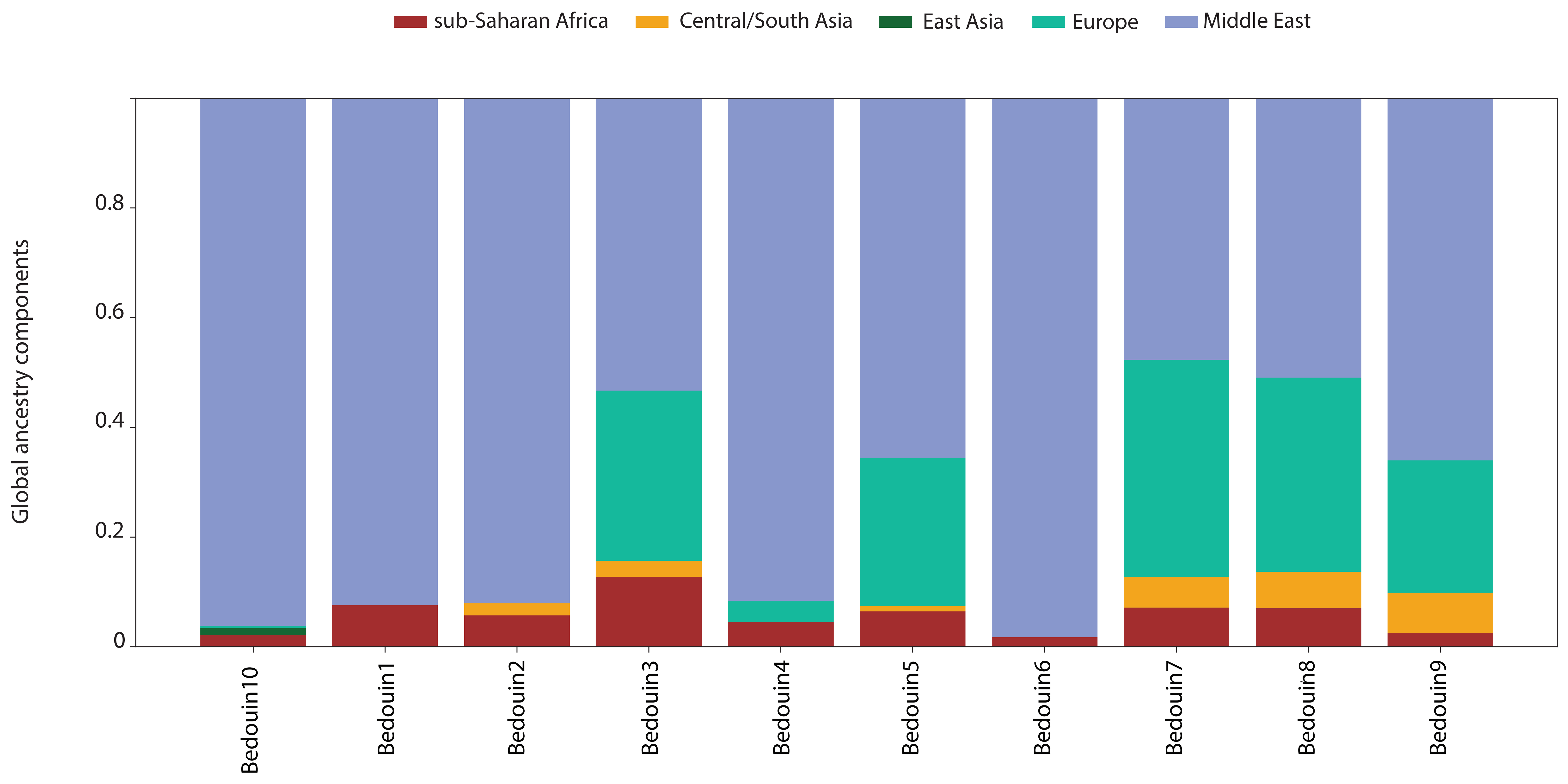
sub-Saharan Africa
Central/South Asia
East Asia
Europe
Middle East
Global ancestry components
0.8
0.6
0.4
0.2
0
Bedouin10
Bedouin1
Bedouin2
Bedouin3
Bedouin4
Bedouin5
Bedouin6
Bedouin7
Bedouin8
Bedouin9

B.

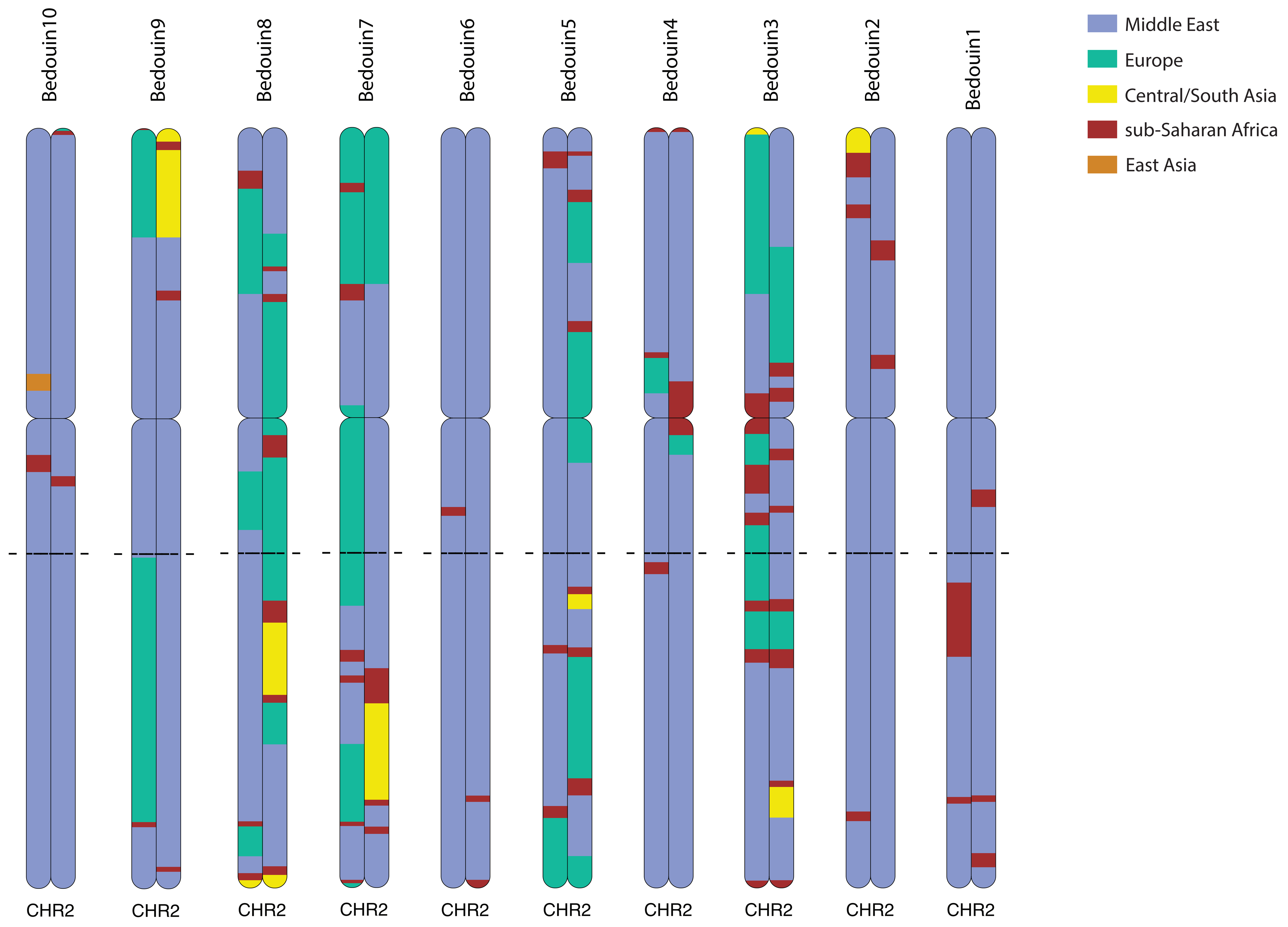
Bedouin10
Bedouin9
Bedouin8
Bedouin7
Bedouin6
Bedouin5
Bedouin4
Bedouin3
Bedouin2
Bedouin1
Middle East
Europe
Central/South Asia
sub-Saharan Africa
East Asia
CHR2

Figure S3
